# Congruence in leaders-subordinates’ mindfulness and knowledge hiding: The role of emotional exhaustion and gender similarity

**DOI:** 10.3389/fpsyg.2022.1007190

**Published:** 2022-10-28

**Authors:** Jun Wan, Zhengqiao Liu, Xianchun Zhang, Xiliang Liu

**Affiliations:** ^1^School of Public Finance and Public Administration, Jiangxi University of Finance and Economics, Nanchang, China; ^2^School of Economics and Management, Yango University, Fuzhou, China; ^3^Maritime Silk Road Tourism Economic Research Center, Guilin Tourism University, Guilin, China; ^4^School of Marxism, Hunan University, Changsha, China

**Keywords:** mindfulness, gender similarity, emotional exhaustion, knowledge hiding, psychological contract

## Abstract

Many scholars have focused on understanding ways of how to suppress knowledge hiding by employees. Existing studies have demonstrated that mindfulness could effectively inhibit employees’ knowledge hiding. This study aims to investigate the impact of leader–subordinate mindfulness congruence on subordinate knowledge hiding and its internal mechanisms. Based on the role theory, we collected 169 leadership data and 368 employee data at three time-points through collecting questionnaire of matching leaders and subordinates. In addition, we used polynomial regression and response surface analysis to validate our research hypotheses. The results demonstrated that: (i) Compared with the “high leader–high subordinate” mindfulness congruence condition, subordinates in the “low leader–low subordinate” mindfulness congruence condition were more likely to exhibit knowledge hiding. (ii) Compared with the “low leader–high subordinate” mindfulness incongruence, subordinates under the “high leader–low subordinate” mindfulness incongruence are more likely to exhibit knowledge hiding. (iii) The more incongruent the mindfulness between the leader and the subordinate is, the more likely an employee is to exhibit knowledge hiding. (iv) Emotional exhaustion mediated the correlation between leader–subordinate mindfulness congruence and knowledge hiding. (v) When the gender of the leader and the subordinate is different, the impact of mindfulness congruence on the inhibition of emotional exhaustion is stronger. This study provides a new perspective for researching the impact of mindfulness on individual behavior and provides a new idea for the research related to inhibiting knowledge hiding.

## Introduction

In the era of knowledge economy, enterprises tend to invest significant time and money in acquiring new knowledge (Zhao et al., 2019) to uphold their competitive advantage. However, some employees are unwilling to share knowledge, and even choose to hide the knowledge which is required by organizational members. Such behavior is referred to as knowledge hiding (Connelly et al., 2012). Knowledge hiding is detrimental to the organization, which can exert a negative impact on the process of knowledge acquisition and organizational performance (Connelly et al., 2012; Černe et al., 2014, 2017). The ways to restrain knowledge hiding exhibited by employees has attracted the attention of many scholars (Gagne et al., 2019; Zhao et al., 2019). The existing studies on this issue mainly have the following limitations. First, compared with the impact of knowledge hiding, a detailed discussion on the antecedents of knowledge hiding was largely overlooked in the existing literatures (Connelly et al., 2012; Zhao et al., 2019). Besides, in the few studies that examined the antecedents of knowledge hiding, researchers mainly focused on the factors that lead to knowledge hiding like workplace bullying and workplace ostracism (Zhao et al., 2016; Zhao and Xia, 2017; Yao et al., 2020a), and paid less attention to inhibitors of knowledge hiding (Jha and Varkkey, 2018; Khalid et al., 2018). From the viewpoint of practice, enhancing inhibitors can be more effective in suppressing the knowledge hiding behavior of employees. Second, the existing research on knowledge hiding mainly focused on colleagues and ignored the knowledge hiding between superiors and subordinates (Zhao and Xia, 2017; Offergelt et al., 2019; He et al., 2020). Although knowledge hiding is more common among colleagues, the ignorance of knowledge hiding between superiors and subordinates may lead to the imperfection of the research system of knowledge hiding. Finally, the existing studies have studied knowledge hiding from the perspective of environmental factors or organizational factors such as the work environment and workplace events, but these factors are primarily uncontrollable (Jha and Varkkey, 2018; Yao et al., 2020a,b). Indeed, restraining employee knowledge hiding through individual factors not only exert a more direct impact but also make the inhibiting effect more continuous once the individual traits are formed. Thus, it is wise for organizations to restrain employee knowledge hiding effectively by excavating individual factors which can restrain employee knowledge hiding such as individual characteristics (Nadeem et al., 2020; Zhao and Jiang, 2021).

**FIGURE 1:**
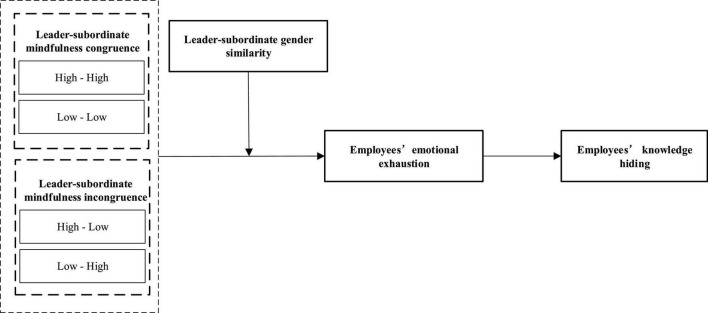
The theoretical model.

Mindfulness, a type of “consciously and objectively paying attention to the existing situation,” can make individuals maintain a clear and calm psychological state (Brown et al., 2007) and focus more on work-related knowledge at work. Meanwhile, mindfulness can promote knowledge-sharing between individuals and organizational members and increases the employees’ emotional resources. thereby enhancing their wellbeing and decreasing turnover intention (Wadlinger and Isaacowitz, 2011; Hulsheger et al., 2013; Simione et al., 2020). When the leaders and their subordinates maintain the same personality, traits, and emotions, they can decrease employees’ emotional resources and enhance their psychological compatibility (Li et al., 2017; Charoensukmongkol and Puyod, 2020). In this way, the employees can fully understand leaders’ wishes and ideas in communication, which can help to attain more tacit cooperation in actions (Li et al., 2017; Charoensukmongkol and Puyod, 2020). In accordance with this logic, leader–subordinate mindfulness congruence, as a matching type, reflects the attention paid by the leader and the subordinate to the current situation. This attention affects both the degree of understanding of the tacit and the degree of concern for the current situation. Meanwhile, it also affects the communication effect and the willingness of sharing knowledge (Coyle and Foti, 2015; Gerpott et al., 2020). Hence, if we want to clarify the correlation between mindfulness and employee knowledge hiding systematically, we need to examine the impact of the leader–subordinate mindfulness congruence on knowledge hiding. Previous studies have reported that mindfulness can markedly inhibit employees’ emotional exhaustion, while emotional exhaustion significantly positively correlates with knowledge hiding (Hulsheger et al., 2013; Simione et al., 2020; Yao et al., 2020a; Zhao and Jiang, 2021). Thus, we propose to use emotional exhaustion as a mediating variable between leader–subordinate mindfulness congruence and knowledge hiding. In addition, previous studies highlighted that leaders with different genders have significantly different influences on the behavior of the subordinates due to differences in gender roles (Pelled and Xin, 1997; Ridgeway, 2001; Richard et al., 2019). If the gender of the leader and the subordinate can meet each other’s need for the role, the consumption of emotional resources of the subordinate can be well alleviated (Cable and Edwards, 2004). Hence, we attempt to use leader–subordinate gender similarity as a boundary condition for the impact of mindfulness congruence on knowledge hiding through emotional exhaustion. Figure 1 shows our research model.

Overall, this study explored the effect mechanism of leader-subordinate mindfulness congruence on subordinate knowledge hiding, with emotional exhaustion as a mediator and gender similarity as a key moderator. We aim to provide guidance for managers on how to reduce employees’ knowledge hiding.

## Theory and hypotheses

### Leader–subordinate mindfulness congruence

Mindfulness initially focused on the individual’s mental clarity and concentration, and then expanded to psychology, neuroscience, and other fields. Many scholars reported that mindfulness is beneficial to the treatment of mental health and other diseases, augment the individual’s wellbeing and life satisfaction, and also exerts an impact on the individual’s attitude and behavior (Bajaj et al., 2016); this provides a new perspective for the research of employee behavior in the field of organizational management. Although the definition of mindfulness has not been unified in the existing literature, the current mainstream definition is proposed by Brown et al. (2007), who highlighted that mindfulness is a trait which strengthens the individual’s attention and awareness of a current event after experiencing this event. Owing to the great potential of mindfulness in the research of employee attitudes and behaviors, many scholars have also discussed the impact of mindfulness (Good et al., 2016).

**FIGURE 2:**
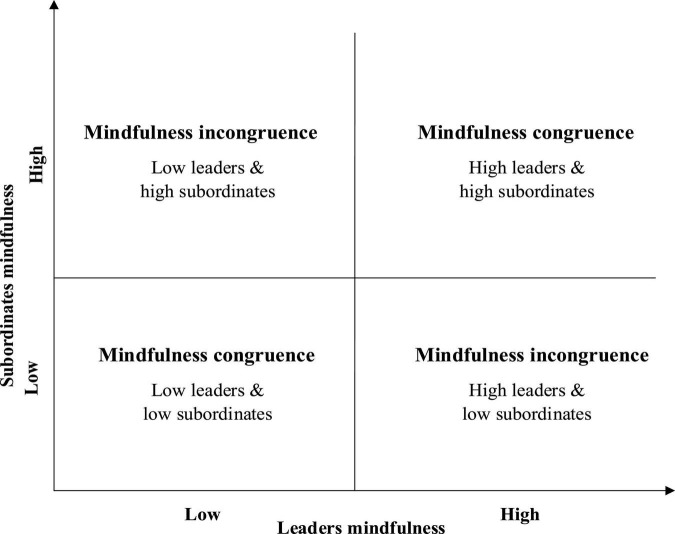
Combinations of leader-subordinate mindfulness.

In an organization, both the leaders and subordinates have their own mindfulness. When the subordinates are more mindful, they tend to feel more respect and support from the leader, and in turn show the willingness to actively communicate with the leader; this can lead to restraining the consumption of their emotional resources and create less knowledge hiding (Hulsheger et al., 2013; Simione et al., 2020; Yao et al., 2020a). Similarly, when the leaders have high mindfulness, they actively communicate with their subordinates, pay attention to subordinates’ needs and provide full support to subordinates; this aids in reducing the consumption of subordinates’ emotional resources and their likelihood of exhibiting negative behaviors (Hulsheger et al., 2013; Li et al., 2017; Yao et al., 2020a). Based on the level of mindfulness of leaders and subordinates, we can combine them into four matching situations (as depicted in Figure 2): high leader–high subordinate (HLHS), low leader–low subordinate (LLLS), high leader–low subordinate (HLLS), and low leader–high subordinate (LLHS). While the first two are mindfulness congruence, the last two are mindfulness incongruence.

### Leader–subordinate mindfulness congruence and knowledge hiding

Role theory highlights that each organizational member plays one or more “roles,” and they will gradually form specific expectations of a particular role in the process of role interaction (Kahn et al., 1964). Role expectation implies the group or individual’s expectation on the characteristics or behaviors of a specific role; this expectation enables the organizational members to quickly enter the role and expect their standardized behavior in the position (Yaffe and Kark, 2011). In an organization, owing to differences in status, power, responsibility, and ability, leaders often act as role senders, while subordinates act as role receivers. If the leaders and the subordinates reach a consensus on the role expectation, they have role consensus. Otherwise, they have discrepancies in role expectation (Graen, 1976; Latack, 1981). Role consensus can not only enhance the individual’s satisfaction and investment in the job role, but also enhance the interpersonal relationship between the two parties (Gross et al., 1958; Matta et al., 2015). In contrast, the role expectation discrepancies will not only damage the relationship between the two parties and induce conflict, but also make both parties experience psychological pain.

In the case of HLHS, leaders and subordinates maintain a high degree of consistency in their attitudes and behaviors toward current events, and they can reach a consensus on their expectations of each other’s roles (Yaffe and Kark, 2011; Matta et al., 2015). First, leaders provide their subordinates with more supportive resources. Leaders and subordinates can maintain a high degree of focus and a more thorough understanding of the current work task. Hence, they can maintain a high degree of consensus in role expectations, which is conducive to improve interpersonal relationship and trust between subordinates and leaders (Eisenbeiss and van Knippenberg, 2015; Pinck and Sonnentag, 2018). Such a high level of trust can inhibit the knowledge hiding behavior of subordinates (Connelly et al., 2012; Bari et al., 2020; Yao et al., 2020b). Second, individuals with a high level of mindfulness can always preserve a calm attitude during completing work tasks; this helps to increase the psychological compatibility between leaders and subordinates while decrease their psychological distance, and enhance the work wellbeing of the subordinates (O’Doherty et al., 2015; Xu et al., 2016; Aguila, 2020). In this case, subordinates strive to meet the leader’s expectations of their role and will less likely to engage in knowledge hiding.

In the case of LLLS, although the leader and the subordinate have reached a consensus in the state of role, the inhibiting effect of this state on knowledge hiding is minimal. As both the leader and the subordinate are unable to focus on the current task, they cannot clearly comprehend this task. Thus, it is tough for the leader to provide the subordinate with any kind of guidance and corresponding supporting resources (Yaffe and Kark, 2011; Ogunfowora, 2014; Matta et al., 2015). Such situation makes the subordinates feel that the leader does not fulfill their role expectations, which in turn leads to the deterioration of the interpersonal relationship between two parties and the subordinates may exhibit knowledge hiding as a response (Connelly et al., 2012; Bari et al., 2020; Yao et al., 2020b). In addition, for individuals with a low level of mindfulness, they can hardly uphold a calm attitude to face various affairs at work, which is not conducive to creating a relaxed and free working environment (O’Doherty et al., 2015; Xu et al., 2016). In this environment, individuals could be more cautious in dealing with the knowledge help from leaders, and thereby tend to engage in knowledge hiding. Based on the above analysis, we propose the following hypothesis:

Hypothesis 1a: Compared with in HLHS, subordinates are more likely to exhibit knowledge hiding in LLLS.

In the case of HLLS, the leader has a relatively high degree of focus on the ongoing task and will learn about the subordinates’ needs through various channels, which not only raises leaders’ expectation on the role of subordinates but also expresses their intention to establish a good interpersonal relationship with the subordinates (Humborstad and Kuvaas, 2013; Binyamin, 2020); this enables the increase of trust between leaders and subordinates, and can well restrain the subordinates’ knowledge hiding. However, the leaders are merely role senders while subordinates are still role receivers and task executors (Connelly et al., 2012; Bari et al., 2020; Yao et al., 2020b). Thus, only if the subordinates have a high level of mindfulness, they can better experience the attention and support given by the leader through a high level of concentration and thereby be more reluctant to hide their knowledge (Eisenbeiss and van Knippenberg, 2015; Pinck and Sonnentag, 2018; Gerpott et al., 2020). When the subordinate have a low level of mindfulness, the role expectation discrepancies between them the their leader will emerge, which may damage the interpersonal relationship and induce role conflict (Humborstad and Kuvaas, 2013). In such scenario, the subordinates will unable to maintain a high level of concentration and they will lack understanding of the current task. Therefore, they cannot feel and understand the support and role expectation given by the leader (Pinck and Sonnentag, 2018; Gerpott et al., 2020; Yao et al., 2020a). When the leader asks for help with knowledge, they will respond to the leader by hiding knowledge (Pinck and Sonnentag, 2018; Gerpott et al., 2020; Yao et al., 2020a).

In the case of LLHS, the role expectations of subordinates are higher than that placed by the leader. In this case, role expectation discrepancies will be easier to form, which can damage the relationship between subordinates and leaders. Leaders with low level of mindfulness lack adequate attention to the task as well as interpersonal interactions, and they will provide limited support and attention to their subordinates (Pinck and Sonnentag, 2018; Aguila, 2020; Gerpott et al., 2020). As employees are the executors and role receivers of the task, those with a higher level of mindfulness can better understand the characteristics of the current task and more actively complete the task assigned by the leader (Nezlek et al., 2016; Tuckey et al., 2018). When the leader asks a subordinate for assistance with knowledge, subordinates are less likely to demonstrate knowledge hiding which may further enlarges the role expectation discrepancies. Hence, we propose the following hypothesis:

Hypothesis 1b: Compared with in LLHS, subordinates are more likely to exhibit knowledge hiding in HLLS.

As mentioned above, four combinations were created according to the different levels of mindfulness of the leader and the subordinate. Among these four combinations, the likelihood of the subordinate to exhibit knowledge hiding from the leader was ranked in the following order: HLHS < LLLS, LLHS < HLLS. We only need to compare any group of HLHS and HLLS, LLLS and LLHS to understand the ranking of the knowledge hiding possibilities of the subordinate in the four combinations, and we selected the second group in this study. In the case of LLLS, they reach the status of role consensus. For employees, their role expectations are satisfied and they have a good emotional experience, thus they eventually work in a good job atmosphere with freedom, pleasure, and a high sense of psychological gain (Aguila, 2020; Charoensukmongkol and Puyod, 2020; Gerpott et al., 2020; Zhao and Jiang, 2021). Although the understanding and concentration of leaders and subordinates on the current tasks are marginally insufficient, when the leader asks the subordinates for knowledge help, the subordinates usually do not deliberately hide it. In the case of LLHS, the role expectation discrepancies causes cognitive disagreements and conflicts between leaders and subordinates in work interactions (Coyle and Foti, 2015). Such disagreements and conflicts consume both leaders and subordinates’ resources to resolve, and thereby make subordinates more cautious in sharing knowledge with leaders (Bari et al., 2020; Gerpott et al., 2020). In addition, in the case of LLLS, the leader and subordinates have a higher degree of tacit understanding with each other, but both lack initiative (Nezlek et al., 2016). While in the case of LLHS, the leader fails to fulfill the role expectations of the subordinates, which leads to the decrease in the degree of trust as well as the frequency of communication between the two parties, but the subordinate may actively shares the knowledge with the leader and is unlikely to practice knowledge hiding (Bari et al., 2020; Gerpott et al., 2020; Yao et al., 2020b). Thus, we consider that in the case of LLLS and LLHS, the likelihood of subordinates to hide knowledge from the leader is similar. Overall, the subordinates were less likely to hide their leaders’ knowledge when mindfulness was congruent compared with when it was incongruent. Hence, we propose the following hypothesis:

Hypothesis 1c: The more incongruent the mindfulness between the leader and the subordinate is, the more likely the subordinate is to exhibit knowledge hiding.

### The mediating effect of emotional exhaustion

Emotional exhaustion is the state and feeling that individual’s emotional resources and related physiological resources are exhausted (Lam et al., 2010; Yao et al., 2020a). To date, several studies have demonstrated a significant positive correlation between individual’s emotional exhaustion and knowledge hiding (Yao et al., 2020a; Ul Ain et al., 2021; Zhao and Jiang, 2021). We propose that the leader–subordinate mindfulness congruence can effectively inhibit the emotional exhaustion of subordinates. First, the mindfulness of the leaders and subordinates enables them to face the current affairs with a calm attitude, and subordinates will be endowed with a better self-regulation ability by mindfulness, thereby making employees more mature in handling emotional management (Walsh and Arnold, 2018; Charoensukmongkol and Puyod, 2020); this enables the subordinates to maintain a good emotional experience and prevent them from falling into a state of emotional exhaustion. Second, the mindfulness of the leaders and subordinates will enable both parties to exhibit a high level of initiative and concentration, which can help the leaders and subordinates have better awareness of themselves and the working environment (especially subordinates). In this situation, the subordinates can better discover and comprehend the leader’s requirements, and the leaders will give more support to the subordinates (Hulsheger et al., 2013; Schuh et al., 2019; Thoroughgood et al., 2020). Moreover, the leaders will affirm and appreciate the efforts of subordinates, which would make subordinates more certain of their value and obtain psychological satisfaction; this can contributes to the enhancement of wellbeing and restrains employees from excessive consumption of their emotional resources at the same time (Bajaj et al., 2016; Xu et al., 2016; Walsh and Arnold, 2020). Finally, the leader–subordinate mindfulness congruence can make both of them reach the state of role consensus and have a higher level of psychological compatibility; this also helps subordinates to gain more robust psychological security, more trust in the leader, and thereby help each other to form a high-quality interpersonal relationship (Aguila, 2020). Hence, besides the conflicts and disagreements in the interaction between superiors and subordinates could be avoid or decrease (Coyle and Foti, 2015), employees can maintain an excellent interpersonal relationship and restrain emotional exhaustion (Li et al., 2017). Thus, the leader–subordinate mindfulness congruence affects the knowledge hiding of subordinates by affecting their emotional exhaustion. Accordingly, we propose the following hypothesis:

Hypothesis 2: Emotional exhaustion mediates the relationship between leader–subordinate mindfulness congruence and knowledge hiding.

### The moderating effect of gender similarity

Gender is a typical demographic feature. Although previous studies have used gender as the control variable of the research model, the differences in the roles between individuals of different genders have been confirmed (Ridgeway, 2001; De Hoogh et al., 2015). For example, females are more likely to be affected by some events, while males are more independent and autonomous (Warren and Catherine, 1999; Lanaj and Hollenbeck, 2015). Owing to the complementarity between individuals of different genders, the disagreements between leaders and subordinates can meet the diversified role needs through role complementarity (Lanaj and Hollenbeck, 2015). To specify, when the gender of the leader and the subordinate is different, the mindfulness of the leader and the subordinate changes their role expectations, and such gender complementarity can better fulfill the expectations of both the leader and the subordinate (Latack, 1981; Ridgeway, 2001). Even if the subordinates fail to fulfill the leader’s expectations, such gender complementarity will help the leader treat the subordinates with a more inclusive attitude, thereby strengthening the subordinates’ trust in the leader (Wang et al., 2013; Kelan, 2020). The satisfaction of role of subordinates expectations and role complementarity can well relieve their emotional resource consumption, thereby better restraining themselves from falling into emotional exhaustion. On contrast, when the leader and the subordinate are of the same gender, although the leader has a higher level of mindfulness, he/she needs to highlight his/her differences between other members of the same gender due to the consideration of their status (Ridgeway, 2001), and tends to focus more on highlighting power distance in their work (Schuh et al., 2014, 2019; Phillips and Grandy, 2018). This leads to the subordinate’ unsatisfied expectations of the leader’s role and increases the psychological distance between them, which hurts the formation of a high-quality interpersonal relationship; thus, it is challenging to restrain the consumption of emotional resources of the subordinates. Accordingly, we propose the following hypothesis:

Hypothesis 3: When the gender of the leader and the subordinate are different, the effect of the mindful congruence on the inhibition of emotional exhaustion is stronger, and thereby inhibits knowledge hiding.

## Materials and methods

### Samples and procedures

Technology enterprises tend to pay more attention to the employees’ knowledge hiding and sharing behavior. In this study, the samples were obtained from Chinese technology enterprises in Shanghai, Guangzhou, and Shenzhen, which is the result of comprehensive consideration of our social network and the degree of fit with the research topic. On the one hand, as the top managers of these enterprises have a good cooperative relationship with the members of the research group, this research has gained their strong support; on the other hand, these technology enterprises are in urgent need of rapid improvement of innovation ability, so they focus more on the knowledge hiding of employees. These practices are common in academic research, e.g., Zhao et al. (2016), Yao et al. (2021), Xu et al. (2022), etc. The research group promised the companies to share the research results and provide free consulting services. Before approaching the target company, we invited eight leaders and eight subordinates to fill the questionnaire, and all of them confirmed that they could understand the listed items very well. The questionnaires were distributed and collected in matching pairs from leaders and subordinates (the questionnaire was filled out anonymously). When the participants filled in the questionnaire, they were in the vicinity of the members of the research group who can address their queries about the item. We checked the filled questionnaires one by one and immediately coded and sealed the questionnaire with an envelope after confirming that there were no missing questions.

To avoid the serious impact of common method variance (CMV) on the research conclusions, we collected questionnaires three times and randomly invited a total of 200 leaders (direct leaders of employees) and 500 subordinates for participation. At time 1, we collected data on mindfulness, demographic variables, and contact information of leaders and subordinates. After 1 month (time 2), we collected data on employees’ emotional exhaustion. After another month (time 3), we collected the data on knowledge hiding by the employees. When the survey was completed, we matched the leaders and subordinates based on the previous code. After eliminating the invalid questionnaires for regular fills and other conditions, we finally obtained 169 effective questionnaires for leaders (effective recovery rate, 84.50%) and 368 questionnaires for employees (effective recovery rate, 73.60%). Among them, the male gender of the subordinate accounted for 50.82%, while the female gender accounted for 49.18%. The average age of subordinates was 30.041 (SD = 5.514) years; 57.34% of subordinates had a graduate degree or above, 42.66% had undergraduate degree or below. The average working time (duration in the company) of subordinates was 5.574 (SD = 5.470) years. The male leaders accounted for 56.21%, while the female leaders accounted for 43.79%. The average age of leaders was 37.142 (SD = 5.653) years, and the average working age (duration in the company) of leaders was 10.243 (SD = 5.237) years.

### Measures

The scales utilized in this study are all relatively mature globally, with good reliability and validity. All scales were translated strictly from English to Chinese following the back-translation procedures by Brislin (1970) to ensure accuracy and internal validity. All items can be viewed in the Supplementary Table 1. The Likert five-point scale (1 = completely disagree; 5 = completely agree) was adopted for the questionnaire. The details are as follows:

#### Leader–subordinate mindfulness congruence

We used the MAAS scale developed by Brown and Ryan (2003) to measure the mindfulness of leaders and subordinates. The original scale had a total of 15 items; however, the item “It seems I am ‘running on automatic’ without much awareness of what I’m doing” did not conform to the Chinese situation, so we removed it. Sample items such as “I could be experiencing some emotion and not be conscious of it until some time later” were scored in reverse.

#### Emotional exhaustion

The emotional exhaustion scale developed by Maslach et al. (2001) was used to measure it, with a total of four items, and sample items such as “I often feel very tired when I get off work.”

#### Knowledge hiding

We referred to the knowledge hiding scale developed by Connelly et al. (2012), with a total of 12 items, and sample items like “when a leader asks me for knowledge help, I will say that I did not know, even though I did.”

#### Leader–subordinate gender similarity

The leader–subordinate gender similarity was measured by dummy variables (subordinates’ gender: 0 = female, 1 = male; Leader’s gender: 0 = female, 1 = male), with 0 = same gender and 1 = different gender.

#### Control variables

Based on the previous studies on knowledge hiding and leader–subordinate congruence (Zhao et al., 2019; Peng et al., 2020; Yao et al., 2020a,b), we took the age, working age, and educational background of the subordinates, as well as the age, working age, and educational background of the leaders, as the control variables in this study.

### Strategic analysis

According to the research hypotheses, we expected that the core variables may not represent a simple linear relationship, so we used polynomial regression to validate the research hypotheses. After centralized all the variables for Hypotheses 1a, 1b, and 1c, we put control variables, leader mindfulness (LM), subordinate mindfulness (SM), squared term of leader mindfulness (LM^2^), product term of leader mindfulness and subordinate mindfulness (LM × SM), and squared term of subordinate mindfulness (SM^2^) into the regression equation to predict emotional exhaustion (EE) and knowledge hiding (KH), as shown in Eq. 1.


(1) $$\begin{array}{l} \text{KH}=a_{0}+a_{1}(LM)+a_{2}(SM)+a_{3}(LM^{2})\\ \;\;\;\;\;\;\;\;\;+a_{4}(LM\times SM)+a_{5}(SM^{2})+e \end{array}$$

Meanwhile, considering the data nesting in this study, the multilevel linear model (HLM) was more suitable for the research hypothesis. Following the advice of Ahearne et al. (2013), we expressed the above formula in layers.


(2) $$Level\text{ 2}:Z_{ij}=\beta_{0j}+\beta_{1j}(LM)+\beta_{2j}(LM^{2})+\gamma_{ij}$$

(3) $$Level\text{ 1}:\beta_{0j}=\gamma_{0}+\gamma_{1}(SM)+\gamma_{2}(SM^{2})+\mu_{0j}$$

(4) $$\beta_{1\,j}=\gamma_{3}+\gamma_{4}\,\left(S\,M\right)+\mu_{1\,j}$$

(5) $$\beta_{2\,j}=\gamma_{5}$$

In the above formulas, γ_0_-γ_*5*_ is equivalent to *a*_*0*_-*a*_*5*_ in Eq. 1, and hypothesis 1a needs to be tested by the value of γ_1_ + γ_2_ on the cross-section of the congruence line (LM = SM). Hypotheses 1b and 1c should be tested by the values of γ_1_−γ_2_ and γ_3_−γ_4_ + γ_5_ on the cross-section of the incongruent line (LM = −SM), respectively. For hypothesis 2, we followed the advice of Edwards and Cable (2009) and multiplied LM, EM, LM^2^, LM × EM, and EM^2^ by their respective regression coefficients and added them to form a BLOCK variable. Thereafter, the bootstrapping test was performed to test the mediating effect of emotional exhaustion. For hypothesis 3, we treated gender similarity as a category variable, and tested the significance of the mediating effect of emotional exhaustion respectively to compare which situation was more significant.

## Results

### Descriptive statistics and correlation analysis

Table 1 shows the mean, standard deviation, and correlation coefficient of each variable. Subordinates’ mindfulness exerted a significant negative effect on emotional exhaustion (*r* = −0.237, *p* < 0.01) and knowledge hiding (*r* = −0.296, *p* < 0.01). Moreover, a significant positive relationship was found between emotional exhaustion and knowledge hiding (*r* = 0.365, *p* < 0.01), which provided preliminary support for the hypotheses.

**TABLE 1:** Mean, standard deviation and correlation coefficient of variables.

Variables	1	2	3	4	5	6	7	8	9	10	11	12	13

Level-1: Subordinates													
(1) Gender	–												
(2) Age	–0.012	–											
(3) Educational background	0.159*	0.126**	–										
(4) Working age	–0.094	0.210**	–0.023	–									
(5) Gender similarity	0.008	–0.037	–0.135	0.044	–								
(6) Mindfulness	0.038	0.157	0.033	0.183*	0.203**	***0.884***							
(7) Emotional exhaustion	−0.154*	–0.073	–0.070	–0.092	−0.235**	−0.237**	***0.913***						
(8) Knowledge hiding	–0.077	0.078	–0.099	0.119	–0.015	−0.296**	0.365**	***0.801***					

**Level-2: Leaders**													

(9) Gender									–				
(10) Age									0.006	–			
(11) Working age									0.063	0.232**	–		
(12) Educational background									–0.148	–0.065	−0.210**	–	
(13) Mindfulness									0.010	–0.052	0.013	0.040	***0.918***
Mean	0.509	30.041	0.574	5.574	0.450	3.588	2.415	2.688	0.497	37.142	10.243	0.562	3.479
SD	0.501	5.514	0.496	5.470	0.499	0.570	0.520	0.470	0.501	5.653	5.237	0.498	0.619

Subordinates *n*_1_ = 368; Leaders *n*_2_ = 169. **p < 0.01, *p < 0.05. The diagonal in bold italics is Cronbach’ α of each variable.

### Reliability and validity

In this study, we used SPSS23.0 software to conduct reliability analysis. Table 1 shows the results. Cronbach α of all variables were >0.8, suggesting the good reliability of the questionnaire. To further determine whether the questionnaire had good discriminative validity, we utilized AMOS23.0 software to conduct the confirmatory factor analysis on variables at the individual level (Table 2). The fitting indexes of the three-factor model was significantly better than other models (χ^2^/df = 1.964, IFI = 0.936, TLI = 0.926, CFI = 0.937, RMSEA = 0.045), suggesting that the three-factor model had good discriminative validity.

**TABLE 2:** Results of confirmatory factor analysis.

Model	factor	χ^2^/df	△χ^2^	IFI	TLI	CFI	RMSEA
Three-factor	SM, EE, KH	1.964	–	0.936	0.926	0.937	0.045
Two-factor	SM + EE, KH	3.561	256.254***	0.883	0.875	0.887	0.064
Two-factor	SM, EE + KH	4.852	460.232***	0.804	0.796	0.812	0.081
Single-factor	SM + EE + KH	6.814	777.042***	0.701	0.695	0.712	0.106

SM, subordinates mindfulness; EE, employees’ emotional exhaustion; KH, employees’ knowledge hiding. ^+^Represents the combination of two factors into one factor. ***p < 0.001.

As subordinates’ mindfulness, emotional exhaustion, and knowledge hiding are all from employees’ self-evaluation, CMV needs to be concerned. Thus, we used Harman single factor test method to test CMV. The results demonstrated that the unrotated first factor accounted for only 30.015% of the total variation, which did not exceed 40%. Meanwhile, Table 2 shows that the fitting indexes of single-factor model were not ideal (χ^2^/df = 6.814, IFI = 0.701, TLI = 0.695, CFI = 0.712, RMSEA = 0.106), suggesting that CMV does not significantly affect the results.

### Hypotheses testing

Before testing hypotheses, we analyzed the distribution of matched sample. The results showed that the sample proportion of leader–subordinate mindfulness congruence, HLLS and LLHS were 40.24, 20.71, and 39.05%, respectively, all were greater than the critical value of 10% required by polynomial regression (Shanock et al., 2010). Therefore, we conducted polynomial regression analysis on the sample data, and Table 3 shows the results.

**TABLE 3:** Polynomial regression and response surface analysis.

Variables	Knowledge hiding				
	
	Model 1	Model 2	Model 3	Response surface	Model 4
**Control variables**					
Subordinate gender	–0.058	–0.021	–0.034		
Subordinate age	0.000	–0.022	–0.018		
Subordinate educational background	–0.083	0.054	0.048		
Subordinate working age	0.003	0.028	0.025		
Leader gender	–0.026	–0.007	–0.020		
Leader age	0.013	0.007	0.007		
Leader educational background	–0.014	0.023	–0.001		
Leader working age	0.006	0.010	0.006		
**Independent variables**					
LM		−0.238***	−0.195**	**Congruence (LM = SM)**	
SM		−0.278***	−0.217***	Slope_1_	−0.412***
LM^2^			0.066*	Curvature_1_	–0.011
LM × SM			−0.095*	**Incongruence (LM = -SM)**	
SM^2^			0.018^+^	Slope_2_	0.022^+^
R^2^	0.255	0.578	0.692	Curvature_2_	0.179*
△R^2^		0.323***	0.114*		

LM, leader mindfulness; SM, subordinate mindfulness. ***p < 0.001, **p < 0.01, *p < 0.05, ^+^*p* < 0.1.

#### Leader–subordinate mindfulness congruence and knowledge hiding

**FIGURE 3:**
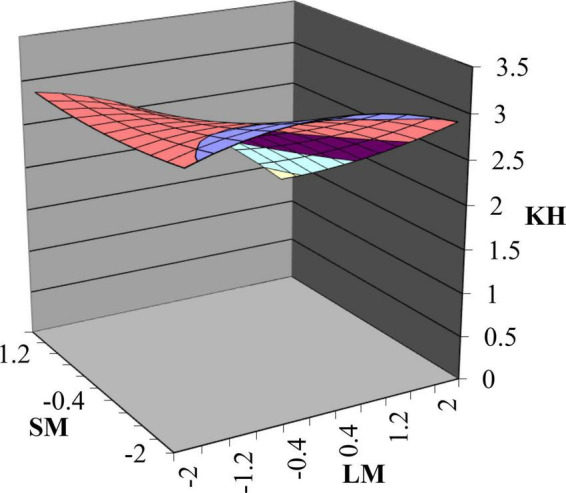
The response surface of leader-subordinate mindfulness congruence and knowledge hiding.

First, Model 3 in Table 3 demonstrates that the change of *R*^2^ increases significantly (Δ*R*^2^ = 0.114, *p* < 0.001), suggesting a non-linear correlation between leader–subordinate mindfulness congruence and subordinate knowledge hiding. Second, Table 3 shows that the curvature of the response surface along the incongruence line (LM = −SM) was significant and >0 (Curvature_2_ = 0.179, *p* < 0.01), suggesting that compared with the leader–subordinate mindfulness congruence, subordinates were more likely to exhibit knowledge hiding under incongruence case, and thereby supporting hypothesis 1c. Third, the slope of the response surface along the congruence line (LM = SM) was significantly < 0 (Slope_1_ = − 0.412, *p* < 0. 001) while the curvature was not significant (Curvature_1_ = − 0.011, *n.s.*), indicating that compared with the case of HLHS, subordinates were more likely to produce knowledge hiding under LLLS, which supported hypothesis 1a. Fourth, the slope of the response surface along the incongruence line (LM = −SM) was significantly positive, indicating that compared with LLHS, subordinates were more likely to produce knowledge hiding under HLLS. Thus, hypothesis 1b is supported. Figure 3 shows the response surface analysis.

#### Mediating effect test

The bootstrap mediating effect test of emotional exhaustion was performed through the PROCESS plug-in of SPSS23.0 software. Table 4 shows that the BLOCK exerted an indirect effect on knowledge hiding [Bia-corrected 95% confidence interval of (0.088, 0.235), excluding 0], indicating that emotional exhaustion exerted a mediating effect between leader–subordinate mindfulness congruence and knowledge hiding. Thus, hypothesis 2 is supported.

**TABLE 4:** Bootstrapping mediation effect test.

Variables	Bootstrapping			
	
	Bia-corrected		Percentile	
	95% CI		95% CI	
		
	Lower	Upper	Lower	Upper
**Direct effect**				

BLOCK → EE → KH	0.278	0.747	0.278	0.747

**Indirect effect**				

BLOCK → EE → KH	0.088	0.235	0.101	0.239

*n*_1_ = 368; *n*_2_ = 169, bootstrapping randomly sampled 20,000 times.

#### Moderating effect test

As the leader–subordinate gender similarity was a category variable, we tested the moderating effect of leader–subordinate gender similarity by categorical testing emotional exhaustion’s mediating effect. Table 5 shows that emotional exhaustion mediated the relationship between mindfulness congruence and knowledge hiding at the 10% level (Model 6, β = 0.118, *p* < 0.1) when the gender of leader–subordinate was the same, while such mediating effect was at the 1% level (Model 3, β = 0.227, *p* < 0.01) when leader–subordinate gender was different. Obviously, compared with the same gender of the leader and subordinate, the emotional exhaustion of the subordinate had a more significant mediating effect in different genders. Thus, hypothesis 3 is supported.

**TABLE 5:** Moderating effect test of leader-subordinate gender similarity.

Variables	Employees’ knowledge hiding					
	
	Leader-subordinate gender difference			Leader-subordinate gender same		
		
	M1	M2	M3	M4	M5	M6
Subordinate age	0.000	–0.009	–0.004	–0.015	–0.033	–0.039
Subordinate educational background	–0.061	0.009	–0.013	–0.039	0.118	0.146
Subordinate working age	0.012	0.026	0.021	0.009	0.033	0.037
Leader age	0.013	0.003	0.003	0.014	0.004	0.007
Leader educational background	0.074	0.095	0.113	–0.093	–0.046	–0.066
Leader working age	0.006	0.012	0.012	0.005	0.017	0.010
Leader-subordinate mindfulness congruence		−0.340***	−0.450***		−0.358**	−0.237**
Employees’ emotional exhaustion			0.227**			0.118^+^
*R* ^2^	0.268	0.408	0.626	0.241	0.594	0.614
△R^2^		0.140***	0.226***		0.353***	0.020^+^

***p < 0.001, **p < 0.01, ^+^*p* < 0.1.

## Discussion

This study explored the influence mechanism of leader-subordinate mindfulness congruence on subordinate knowledge hiding. In fact, the means to restrain the knowledge hiding of subordinates has always been the focus of many scholars and managers (Zhao et al., 2019; Yao et al., 2020a,b). Previous studies have established that mindfulness can effectively promote subordinates to actively share knowledge (Gerpott et al., 2020). Nevertheless, it is still unclear what kind of situations is best suited to inhibit knowledge hiding when considering mindfulness in both leaders and subordinates. Based on role theory, we collected 169 leadership data and 368 employee data. The results demonstrated that: (i) compared with the HLHS, the subordinates were more likely to exhibit knowledge hiding under LLLS; (ii) compared with the LLHS, the subordinates were more likely to exhibit knowledge hiding under HLLS; (iii) the more incongruent the mindfulness between the leader and the subordinate is, the more likely the employee is to exhibit knowledge hiding; (iv) emotional exhaustion mediated the relationship between leader–subordinate mindfulness congruence and knowledge hiding; (v) when the gender of the leader and the subordinate were different, the effect of mindfulness congruence on the inhibition of emotional exhaustion was stronger. In conclusion, our study shows that leader-subordinate mindfulness congruence has an important impact on subordinates’ emotional exhaustion and knowledge hiding, and leader-subordinate gender similarity plays an important role in the above relationship. In the following section, we discuss the theoretical and practical implications of this study.

### Theoretical implications

First, we focused on the impact of mindfulness of both leaders and subordinates on the subordinates’ knowledge hiding. Previous studies on mindfulness only selected leaders or subordinates as a single perspective to analyze the effect on outcome variables, while ignored the “resultant force” of leaders–subordinates’ mindfulness and lacked the comparative studies of different combinations (Eisenbeiss and van Knippenberg, 2015; Pinck and Sonnentag, 2018; Schuh et al., 2019; Gerpott et al., 2020). In addition, the existing literature paid more attention to the impact of mindfulness on individual wellbeing, job performance, turnover intention, and job satisfaction (Brown and Ryan, 2003; Dane and Brummel, 2014; Bajaj et al., 2016; Phillips and Grandy, 2018; Simione et al., 2020), while overlooked the crucial role of mindfulness in the employee knowledge flow process (Gerpott et al., 2020). Therefore, we shifted our research perspective to the bilateral perspective of “leaders and subordinates.” On the one hand, we verified the crucial role of different leader–subordinate mindfulness combinations in inhibiting the subordinate’s knowledge hiding. On the other hand, we unearthed which combination of leader–subordinate mindfulness can inhibit subordinates’ knowledge hiding most effectively. Furthermore, unlike previous studies on leader–subordinate matching, we believe that it is not always better if the subordinate has higher ability (Humborstad and Kuvaas, 2013; Peng et al., 2020), instead, to truly influence the subordinate’s behavior, the cooperation of the leader is required. Thus, this study provides a new perspective for studying impact of mindfulness on individual behavior, and offers a new idea for the related research of inhibiting knowledge hiding.

Second, we found a bridge between subordinates’ mindfulness and knowledge hiding. There has been considerable research on the impact of mindfulness on employee’s emotional exhaustion (Hulsheger et al., 2013; Eisenbeiss and van Knippenberg, 2015; Li et al., 2017; Charoensukmongkol and Puyod, 2020). However, as a variable trait of individuals, mindfulness is easily affected by individual’s psychological states and emotions, and the correlation between mindfulness and changes in individual emotional resources has always been a crucial research direction in the field related to mindfulness (Eisenbeiss and van Knippenberg, 2015; Li et al., 2017; Walsh and Arnold, 2018). Therefore, whether the change of individual emotional resources can play a mediating role in the correlation between mindfulness congruence and knowledge hiding requires further verification. By validating the mediating role of employee emotional exhaustion, we can effectively supplement the relevant studies and confirm the crucial role of employee emotional exhaustion in the field of mindfulness and knowledge hiding (Yao et al., 2020a; Zhao and Jiang, 2021).

Finally, we included gender as a crucial boundary condition in the leader–subordinate matching research. The leader-member exchange theory has demonstrated a complex relationship between leaders and subordinates. Many scholars examined the factors affecting the relationship between leaders and subordinates, among which, gender stands as one of the most crucial factors (Latack, 1981; Ridgeway, 2001). However, most existing studies used gender as a control variable to analyze individual behavior (Yao et al., 2020a,b; Zhao and Jiang, 2021), while ignored the crucial role of the gender combination of leaders and subordinates in the theoretical model. We used the leader–subordinate gender similarity as the boundary condition between mindfulness congruence and emotional exhaustion, and established that when the leader and the subordinate have different genders, the effect of mindfulness congruence on individual emotional exhaustion is stronger and the subordinate is less likely to exhibit knowledge hiding. This finding not only confirms the complementarity principle in role theory but also finds a demographic gender explanation for the inhibition mechanism of knowledge hiding (Lanaj and Hollenbeck, 2015).

### Practical implications

First, in the recruitment process, the organization should include the level of mindfulness of the applicants in the evaluation index system, because individuals with a high level of mindfulness can better inhibit knowledge hiding. Meanwhile, in the promotion process, besides evaluate their ability, it is also necessary to assess their level of mindfulness (Brown and Ryan, 2003; Bajaj et al., 2016; Phillips and Grandy, 2018; Simione et al., 2020) since leaders with a high level of mindfulness can help subordinates to relieve the consumption of emotional resources and inhibit knowledge hiding.

Second, the organization also needs to actively execute training to improve the level of mindfulness of organizational members, such as mindfulness stress reduction method and cognitive therapy (Brown et al., 2007; Bajaj et al., 2016; Aguila, 2020; Simione et al., 2020). If the organization has enough resources, it needs to systematically increase the level of mindfulness of all organizational members including leaders and subordinates. When the resources are limited, the organization should focus more on improving subordinates’ level of mindfulness, and thereby help the organization to alleviate the negative effects caused by leader–subordinate mindfulness incongruence to some extent.

Third, organizations need to help employees supplement emotional resources in time to avoid employees falling into emotional exhaustion (Lee and Chelladurai, 2016; Xu et al., 2018). For example, when employees are depressed, leaders should provide care and encouragement, establish places for emotional catharsis and build a caring mechanism for employees (Yao et al., 2020a).

Finally, organizations can arrange gender-complementary leaders to form stable leader–subordinate relationships with subordinates which are limited to work (Lanaj and Hollenbeck, 2015); this also helps to relieve the emotional resource consumption of subordinates and thus inhibit the subordinates’ knowledge hiding more effectively.

### Limitations and future research directions

This study has a few limitations. First, none of the used scales were developed in China. Although we strictly followed the translation and translation back procedures to decrease errors and the scale showed good reliability and validity, there could still be some limitations when applied to Chinese situations directly. In future, we will develop localized scales to improve the applicability (Brown and Ryan, 2003; Yao et al., 2020a,^2021^; Xu et al., 2022).

Second, although we collected data in three time points, the measurements of subordinates’ mindfulness, emotional exhaustion, and knowledge hiding all come from the same subject, which inevitably leads to CMV (Malhotra et al., 2006; Spector, 2006). Thus, future studies should utilize a combination of self-evaluation and evaluation of others to measure these variables.

Third, although we integrated gender as demography variables into our research model, there remain many other demographic variables that can be introduced into the organizational behavior, such as similarity in educational background, age similarity, marital status similarity, and number of children similarity, these variables could be taken into account in future research (Brouer et al., 2009; Abu Bakar and McCann, 2014).

Fourth, based on the cognitive affective personality system theory, the consumption of emotional resources is always accompanied by the change of individual cognition. Thus, future studies can include cognitive variables such as relational identification and organizational identification in the theoretical framework (Yao et al., 2020a,b). In addition, the research on leader–subordinate matching includes the interaction between leaders and subordinates, during which some resources might be exchanged. Hence, further research can also try to incorporate leader-member exchange into the research framework (Zhao et al., 2019).

## Conclusion

This study aims to explore how leaders can suppress subordinates’ knowledge hiding, thereby helping managers better suppress such negative behavior. Our study found that subordinates were more likely to exhibit knowledge hiding under LLLS and HLLS situation; the more incongruent the mindfulness of leaders-subordinates is, the more likely the employees are to exhibit knowledge hiding; subordinates’ emotional exhaustion plays a mediating role between leader-subordinate mindfulness congruence and knowledge hiding. When the gender of leaders and subordinates are different, mindfulness congruence has a stronger inhibitory effect on emotional exhaustion, and thus inhibits knowledge hiding. Overall, leader-subordinate mindfulness congruence exerts an impact on employees’ knowledge hiding through emotional exhaustion, and gender similarity could strengthen the aforementioned effects of leader-subordinate mindfulness congruence. Our findings comprehend the understanding of knowledge hiding and leader-subordinate mindfulness by focusing on the role theory and provide several suggestions that managers can follow to prevent, control, and reduce employees’ knowledge hiding.

## Data availability statement

The raw data supporting the conclusions of this article will be made available by the authors, without undue reservation.

## Author contributions

JW conceived and designed the study, and completed the manuscript in English. ZL participated in drafting the manuscript and revised it critically for critical intellectual content. XZ and XL gave many good research advices and revised the manuscript. All authors contributed to the article and approved the submitted version.
