# Supplementary material for: Congruence in leaders-subordinates’ mindfulness and knowledge hiding: The role of emotional exhaustion and gender similarity
Source: Front Psychol. 2022 Oct 28;13:1007190. doi: 10.3389/fpsyg.2022.1007190 (PMC9650406; doi:10.3389/fpsyg.2022.1007190)
Supplement: Supplementary file 1 [file Table_1.DOCX]

**Section A: Mindfulness**

| A1 I could be experiencing some emotion and not be conscious of it until some time later. | 我可能正在经历一些情绪上的事情，但要到一段时间后才能意识到。 |
| --- | --- |
| A2 I break or spill things because of carelessness, not paying attention, or thinking of something else. | 我打碎或打翻东西是因为我不小心、不注意或者在想别的事情。 |
| A3 I find it difficult to stay focused on what’s happening in the present. | 我发现我很难把注意力集中在目前正在发生的事情上。 |
| A4 I tend to walk quickly to get where I’m going without paying attention to what I experience along the way. | 我倾向于快速步行去我要去的地方，而不注意沿途的经历。 |
| A5 I tend not to notice feelings of physical tension or discomfort until they really grab my attention. | 我往往不会注意到身体紧张或不适的感觉，直到我不得不注意到它们。 |
| A6 I forget a person’s name almost as soon as I’ve been told it for the first time. | 我一般在第一次被人告诉我某人的名字时候就把这个名字给忘了。 |
| A7 It seems I am “running on automatic” without much awareness of what I’m doing. | 我似乎是在“自动运行”，没有意识到我在做什么。 |
| A8 I rush through activities without being really attentive to them. | 我完成活动都很匆忙以至于没有真正注意到它们。 |
| A9 I get so focused on the goal I want to achieve that I lose touch with what I am doing right now to get there. | 我太专注于我想要实现的目标了，以至于我忽略了我现在正在做的事情。 |
| A10 I do jobs or tasks automatically, without being aware of what I’m doing. | 我一般在完成工作或任务时都是无意识的。 |
| A11 I find myself listening to someone with one ear, doing something else at the same time. | 我发现自己经常一边听别人说话，一边做着别的事情。 |
| A12 I find myself preoccupied with the future or the past. | 我发现自己专注于未来或过去。 |
| A13 I find myself doing things without paying attention. | 我发现自己做事时总是心不在焉。 |
| A14 I snack without being aware that I'm eating. | 我没有意识到我在吃东西。 |

**Section B: Emotional exhaustion**

| B1 I feel emotionally drained from my work. | 我觉得工作让我的情绪枯竭了。 |
| --- | --- |
| B2 I often feel very tired when I get off work. | 我下班后经常感到很累。 |
| B3 When I get up in the morning, I feel tired at the thought of facing a new day at work. | 当我早上起床时，一想到要面对新的一天的工作，我就感到很累。 |
| B4 I feel like I’m at the end of my rope. | 我觉得我已经无计可施了。 |

**Section C: Knowledge hiding**

| C1 Agreed to help him/her but never really intended to. | 虽然同意帮助他/她，但并不是真的愿意。 |
| --- | --- |
| C2 Told him/her that I would help him/her out later but stalled as much as possible. | 跟他/她说我晚一点会帮他/她，但是尽量拖延。 |
| C3 Looked into the request to make sure my answers were accurate. | 仔细考虑这个请求以确保我的回答是准确的。 |
| C4 Explained everything very thoroughly. | 把一切都解释清楚。 |
| C5 Answered all his/her questions immediately. | 立即回答他/她所有的问题。 |
| C6 Told my coworker exactly what s/he needed to know. | 告诉我的同事他/她需要知道的事情。 |
| C7 Pretended that I did not know the information. | 假装我不知道这个信息。 |
| C8 Said that I did not know, even though I did. | 即使我知道我也会说我不知道。 |
| C9 Pretended I did not know what s/he was talking about. | 假装我不知道她/他在说什么。 |
| C10 Explained that I would like to tell him/her, but was not supposed to. | 解释说我想但是不能告诉他/她。 |
| C11 Explained that the information is confidential and only available to people on a particular project. | 解释说，这些信息是保密的，只对参与特定项目的人开放。 |
| C12 Told him/her that my boss would not let anyone share this knowledge. | 告诉他/她，我的老板不会让任何人分享这些信息。 |
